# AMFF-Net: An Adaptive Multi-Layer Feature Fusion Network Based on ConvNeXtV2-B for Medical Image Classification

**DOI:** 10.3390/bioengineering13090983

**Published:** 2026-08-26

**Authors:** Min-Seo Kim, Hyoung-Gook Kim

**Affiliations:** Department of Electronic Convergence Engineering, Kwangwoon University, 20 Gwangun-ro, Nowon-gu, Seoul 01897, Republic of Korea; messiminseo@kw.ac.kr

**Keywords:** medical image classification, ConvNeXtV2-B, hierarchical feature fusion, efficient channel attention

## Abstract

Although ConvNeXtV2 has shown promising performance in medical image classification, approaches relying primarily on final-stage features may underutilize low-level structural and complementary hierarchical information. To address this limitation, we propose an Adaptive Multi-Layer Feature Fusion Network (AMFF-Net) based on ConvNeXtV2-B for medical image classification. The proposed framework employs a Feature Alignment (FA) module to project multi-stage features into a unified representation space and an Adaptive Multi-Layer Feature Fusion (AMFF) module to compute a single input-dependent scalar weight for each stage and dynamically adjust the relative contributions of hierarchical features. An Efficient Channel Attention (ECA) module is subsequently incorporated to enhance the fused representation through lightweight channel-wise recalibration. AMFF-Net was evaluated on three medical image classification datasets: Kvasir-v2, HAM10000, and ChestXray14. Experimental results demonstrate that AMFF-Net consistently improves classification performance over the baseline ConvNeXtV2-B and achieves competitive performance compared with representative convolutional neural network (CNN)- and Transformer-based architectures, while incurring relatively modest additional computational overhead. Ablation results further support the contribution of FA, AMFF, and ECA to the overall performance of the proposed framework.

## 1. Introduction

Medical image classification is a fundamental technology for computer-aided diagnosis (CAD), disease prediction, and clinical decision support systems. Recent advances in deep learning have significantly improved various medical image analysis tasks, including X-ray, computed tomography (CT), magnetic resonance imaging (MRI), fundus, and dermoscopic image classification [1]. Unlike conventional approaches based on handcrafted features, deep neural networks can automatically learn discriminative representations from large-scale medical image datasets, leading to improved diagnostic performance [2].

Early deep learning-based medical image analysis primarily relied on convolutional neural networks (CNNs) [2], which effectively learned local structural features through hierarchical representations. Subsequently, transformer-based models [3] improved feature representation by capturing long-range dependencies and global contextual information through the self-attention mechanism. More recently, ConvNeXt [4] combined the computational efficiency of CNNs with Transformer-inspired representation capability, and ConvNeXtV2 has demonstrated strong potential as an effective backbone for medical image analysis owing to its enhanced feature representation capability and favorable computational efficiency [5].

However, existing deep learning-based medical image classification models still face limitations in fully utilizing hierarchical feature information. Many networks primarily rely on high-level semantic features extracted from the final network stage, which may result in the insufficient utilization of fine-grained structural details captured in earlier stages. Because medical images contain important diagnostic cues, such as lesion boundaries, tissue textures, and morphological variations, effectively integrating multi-level features can help improve classification robustness and discriminative capability.

Furthermore, medical images exhibit significant variations in lesion size, location, and morphology across samples, which may limit the ability of fixed feature fusion strategies to adapt to diverse image characteristics. Therefore, an adaptive feature fusion framework can provide a mechanism for selectively exploiting complementary information from multi-level features while preserving both spatial details and high-level semantic representations.

To address these limitations, this paper proposes an Adaptive Multi-Layer Feature Fusion Network (AMFF-Net) based on ConvNeXtV2-B. The proposed framework integrates multi-stage features through Feature Alignment (FA) and Adaptive Multi-Layer Feature Fusion (AMFF), followed by lightweight channel recalibration using Efficient Channel Attention (ECA), while introducing modest computational overhead. Through this integrated framework, AMFF-Net aims to enhance the utilization of complementary hierarchical representations for medical image classification.

The main contributions of this paper can be summarized as follows:

First, we propose an integrated hierarchical feature fusion framework that combines Feature Alignment (FA) and Adaptive Multi-Layer Feature Fusion (AMFF) to exploit multi-stage features extracted from ConvNeXtV2-B. FA aligns multi-stage features into a common representation space, while AMFF performs input-dependent stage-level adaptive fusion by computing a normalized scalar weight for each network stage. Importantly, the adaptive weighting is performed at the stage level, with a single input-dependent weight assigned to each stage rather than separate weights for individual channels or spatial locations.

Second, we incorporate an Efficient Channel Attention (ECA) module as a lightweight post-fusion channel recalibration mechanism within the proposed framework. By emphasizing informative feature channels, ECA enhances the discriminative representation of the fused features while maintaining computational efficiency. Importantly, ECA serves as a complementary component of the proposed framework rather than as an independent methodological contribution.

Third, we evaluate the effectiveness of the proposed AMFF-Net on three medical image classification datasets, Kvasir-v2 [6], HAM10000 [7], and ChestXray14 [8], representing gastrointestinal endoscopy, skin lesion, and chest X-ray imaging, respectively. AMFF-Net achieves 96.72% accuracy on Kvasir-v2, 95.25% accuracy on HAM10000, and 89.86% macro area under the receiver operating characteristic curve (AUC) on ChestXray14. Comparative experiments with representative CNN- and Transformer-based architectures, including ConvNeXt, ConvNeXtV2, EfficientNetV2, and Vision Transformer, together with ablation studies, demonstrate the effectiveness of the proposed integrated framework and the contribution of its constituent components.

The remainder of this paper is organized as follows. Section 2 reviews related work on medical image classification, hierarchical and adaptive feature fusion, and channel attention mechanisms. Section 3 presents the proposed AMFF-Net architecture and its main components. Section 4 describes the datasets, experimental setup, and evaluation metrics, and presents the comparative, ablation, computational-complexity, and qualitative results. Section 5 discusses the main findings and limitations of the study. Finally, Section 6 concludes the paper and outlines directions for future work.

## 2. Related Work

### 2.1. CNN- and Transformer-Based Medical Image Classification

Recent advances in deep learning have significantly improved medical image classification. CNN-based architectures, including ResNet [9], DenseNet [10], and EfficientNet [11], effectively capture local structural features, whereas Transformer-based models, such as ViT [12] and Swin Transformer [13], capture global contextual information through self-attention. More recently, ConvNeXt incorporated Transformer-inspired designs into CNNs, and ConvNeXtV2 further enhanced feature representation and generalization through Global Response Normalization (GRN) [5] and Masked Autoencoder (MAE)-based self-supervised learning [14], making it a promising backbone for medical image analysis.

Nevertheless, many existing ConvNeXtV2-based classification approaches primarily rely on high-level semantic representations from the final network stage, potentially leaving complementary structural information from earlier stages underutilized. Consequently, effectively exploiting hierarchical feature representations remains an important research issue in ConvNeXtV2-based medical image classification.

### 2.2. Hierarchical and Adaptive Feature Fusion

Hierarchical feature fusion has been extensively investigated to exploit complementary information from different stages of deep neural networks [15,16]. Early stages capture low-level structural information with high spatial resolution, whereas deeper stages provide high-level semantic representations with stronger discriminative capability. Therefore, integrating features across stages can provide richer representations than relying solely on the final-stage features. Representative approaches include Feature Pyramid Network (FPN) [17], Path Aggregation Network (PANet) [18], and Bidirectional Feature Pyramid Network (BiFPN) [19], which effectively integrate multi-scale features for object detection. Similar multi-stage feature aggregation has also been applied to medical image analysis, as demonstrated by multi-skip architectures such as UNet++ [20].

Although these approaches effectively integrate multi-scale features, many were originally developed for object detection or segmentation. In medical image classification, hierarchical feature fusion has also been explored using relatively simple schemes, such as feature concatenation, element-wise addition, or other predetermined operations [21,22,23]. These approaches typically rely on fixed aggregation rules or input-independent learnable stage weights, so the relative contribution of each stage does not explicitly vary across input images. In contrast, input-dependent fusion dynamically adjusts the relative importance of each feature stage according to image-specific characteristics.

To enable multi-stage fusion, feature alignment operations such as channel projection and spatial alignment reconcile differences in channel dimensions and spatial resolutions. These operations serve as enabling mechanisms for multi-stage feature fusion rather than constituting the adaptive fusion mechanism itself.

### 2.3. Channel Attention Mechanisms

In addition to hierarchical feature fusion, attention mechanisms have been widely adopted to enhance feature representations by emphasizing informative features and suppressing redundant information. Representative channel attention methods include Squeeze-and-Excitation Network (SE-Net) [24], Convolutional Block Attention Module (CBAM) [25], and Efficient Channel Attention Network (ECA-Net) [26]. SE-Net models channel interdependencies using Global Average Pooling (GAP) and fully connected layers, while CBAM combines channel and spatial attention. ECA-Net provides lightweight channel attention using one-dimensional convolution without channel dimensionality reduction, thereby reducing computational complexity.

Although channel attention effectively enhances feature representations, it primarily recalibrates channel responses within individual feature representations rather than explicitly modeling complementary information across multiple network stages. Therefore, channel attention serves as a complementary refinement mechanism after hierarchical feature fusion rather than a substitute for stage-level feature fusion.

The key methodological distinction of AMFF-Net lies in integrating multi-stage ConvNeXtV2-B representations through input-dependent stage-level adaptive fusion, followed by lightweight channel recalibration, rather than in introducing feature alignment or channel attention as individual components. Specifically, FA serves as an enabling operation that aligns multi-stage features through channel projection and spatial alignment, while AMFF constitutes the core fusion mechanism by assigning a single input-dependent scalar weight to each aligned stage. ECA then performs lightweight post-fusion channel recalibration. Thus, AMFF-Net integrates these complementary operations to exploit hierarchical representations, with AMFF adapting the relative contribution of each stage to the input image.

## 3. Proposed Method

### 3.1. Overview of the Proposed AMFF-Net

**Figure 1:**
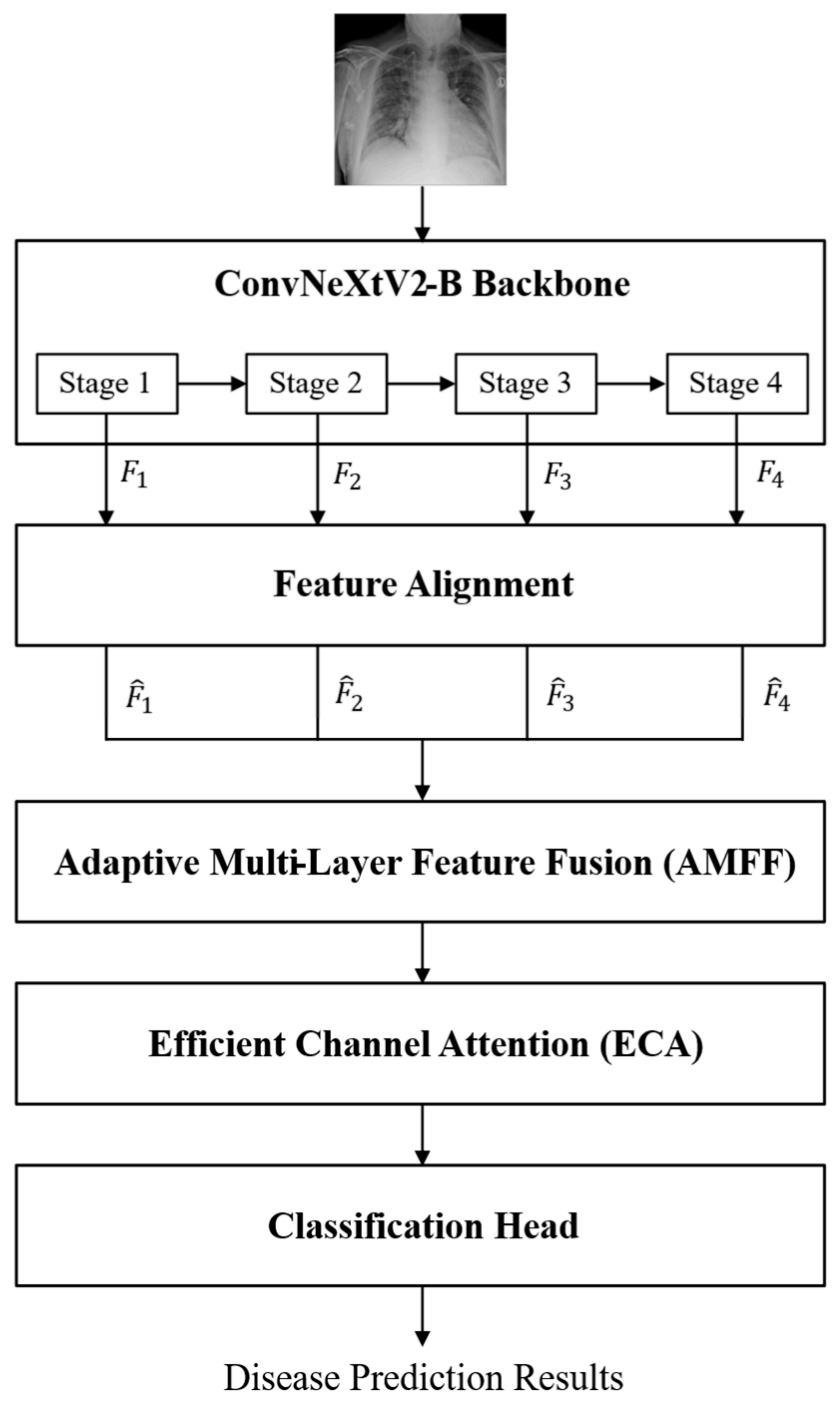
Overall architecture of the proposed network.

As illustrated in Figure 1, the input medical image is sequentially processed through the four stages of ConvNeXtV2-B, producing hierarchical feature maps F1, F2, F3, and F4. The earlier stages primarily capture low-level structural information, such as lesion boundaries, textures, and local shapes, whereas the later stages capture more abstract semantic representations related to lesion morphology and disease categories [27,28,29]. However, the features extracted from different stages differ in spatial resolution, channel dimension, and semantic representation level. Therefore, directly fusing these features may lead to dimensional inconsistencies and feature mismatches, making effective feature integration difficult [30]. To address this issue, the FA module aligns the features extracted from the individual stages within a common feature representation space. Specifically, channel projection and spatial resolution alignment transform the hierarchical features into a common representation with compatible channel dimensions and spatial resolutions, thereby facilitating subsequent hierarchical feature fusion [17,30].

Subsequently, the aligned features are passed to the AMFF module. AMFF utilizes the global feature representation of each stage to estimate a single input-dependent scalar weight and dynamically adjusts the relative contribution of each stage based on the features obtained from the input medical image. The resulting stage-level weight is applied uniformly to all channels and spatial locations within the corresponding stage feature. Thus, AMFF performs adaptive fusion exclusively at the stage level, rather than applying separate adaptive weights to individual channels or spatial locations.

The fused feature is subsequently recalibrated at the channel level using the ECA module. ECA performs lightweight channel-wise recalibration by enhancing informative channels and suppressing less relevant responses, thereby refining the discriminative representation of the fused features while introducing limited computational overhead.

Finally, the refined feature is fed into the classification head to predict the disease class of the input medical image.

### 3.2. Hierarchical Feature Extraction Using ConvNeXtV2-B

In this study, ConvNeXtV2-B is employed as the backbone network to extract hierarchical features with different levels of abstraction from input medical images. ConvNeXtV2 preserves the local feature extraction capability of convolutional neural networks while incorporating large-kernel depthwise convolutions, an inverted bottleneck structure, and Global Response Normalization (GRN), thereby providing a large receptive field and enhanced feature representation capability. In particular, GRN adjusts features based on their global channel responses, alleviating excessive information concentration in specific channels and improving channel-wise feature diversity.

**Figure 2:**
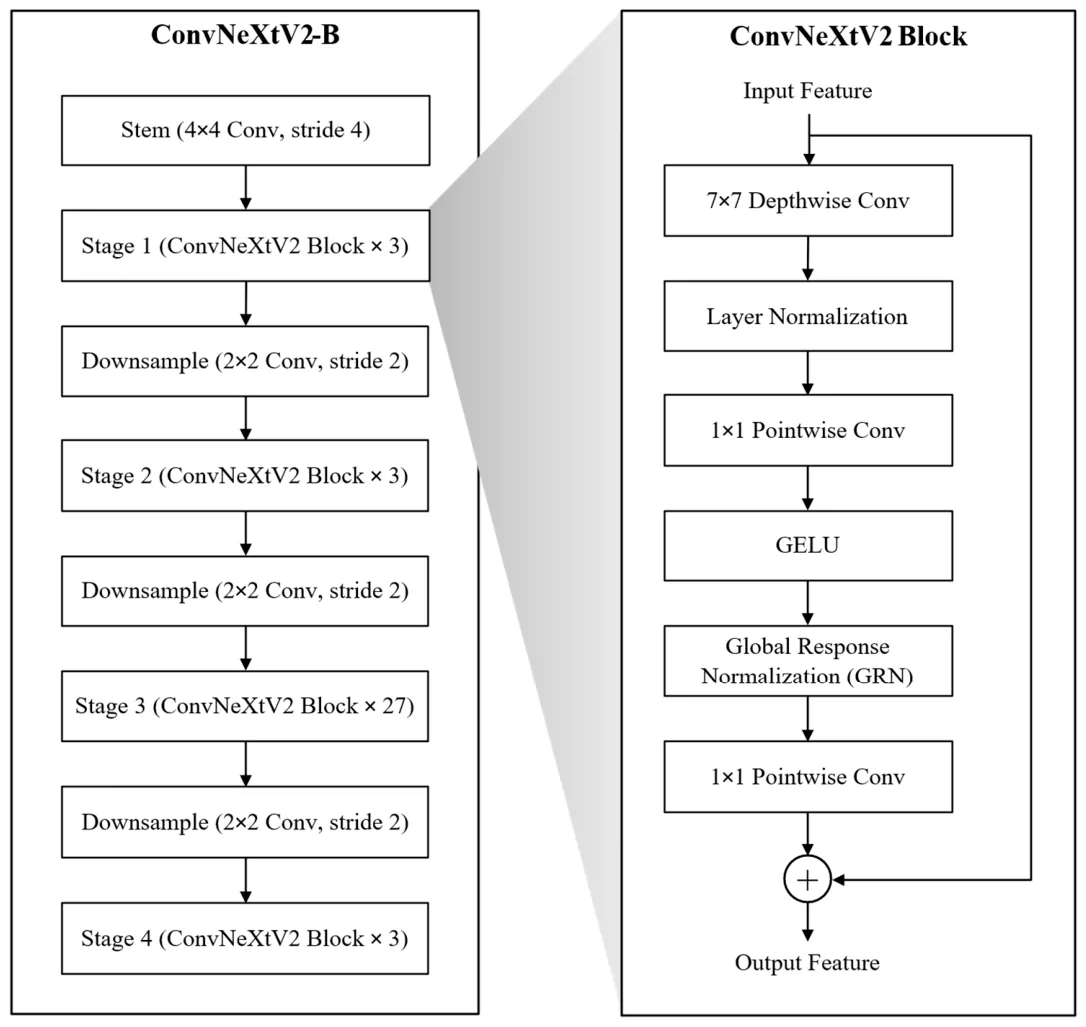
Architecture of the ConvNeXtV2-B backbone and ConvNeXtV2 block.

Figure 2 illustrates the overall architecture of ConvNeXtV2-B and the ConvNeXtV2 block that constitutes each stage. ConvNeXtV2-B consists of a stem, four stages, and three downsampling layers positioned between adjacent stages. The input medical image is represented as follows:

(1) $$X\in R^{H\times W\times 3},$$

where H and W denote the height and width of the input image, respectively, and 3 denotes the number of input channels. The input image is transformed into the initial feature representation $X_{s}$ through the stem and then sequentially propagated from Stage 1 to Stage 4. The four stages of ConvNeXtV2-B consist of 3, 3, 27, and 3 ConvNeXtV2 blocks, respectively. The output of each stage is defined as follows:

(2) $$F_{i}=\left\{\begin{array}{c} S_{1}\left(X_{s}\right),\;\;i=1,\\ S_{i}\left(D_{i-1}\left(F_{i-1}\right)\right),\;\;i\in \left\{2,\;3,\;4\right\}, \end{array}\;\;X_{s}=Stem\left(X\right)\;\right.,$$

where $S_{i}\left(\cdot \right)$ denotes the ConvNeXtV2 block operations in the i-th stage, and $D_{i-1}\left(\cdot \right)$ represents the downsampling operation between adjacent stages. Each downsampling layer uses a 2 × 2 convolution with a stride of 2 to reduce the spatial resolution by half while increasing the number of channels.

Each ConvNeXtV2 block consists of a 7 × 7 depthwise convolution, layer normalization, two 1 × 1 pointwise convolutions, Gaussian Error Linear Unit (GELU) activation, GRN, and a residual connection [31,32,33,34,35].

The 7 × 7 depthwise convolution efficiently extracts spatial information over a large receptive field, whereas the two pointwise convolutions transform the channel dimension according to C→4C→C, thereby facilitating inter-channel feature interaction. GRN normalizes the global response of each channel to improve feature diversity, and the residual connection combines the transformed features with the original input features.

The feature map generated by the i-th stage is represented as follows:

(3) $$F_{i}\in R^{H_{i}\times W_{i}\times C_{i}},\;\;i\in \left\{1,\;2,\;3,\;4\right\},$$

where $H_{i}$, $W_{i}$, and $C_{i}$ denote the height, width, and number of channels of the i-th stage feature, respectively. As the network depth increases, the spatial resolution of the feature maps progressively decreases, whereas the number of channels and the level of semantic abstraction increase. Accordingly, Stage 1 primarily extracts low-level structural features, such as lesion boundaries and textures, whereas Stage 2 learns intermediate-level representations that capture detailed structures and partial lesion morphology. Stage 3 captures the overall lesion morphology and contextual information from the surrounding tissues owing to its larger receptive field, whereas the final Stage 4 generates the high-level semantic representations required for classification.

The complete set of hierarchical features generated by ConvNeXtV2-B is defined as follows:

(4) $$F=\left\{F_{1},\;F_{2},\;F_{3},\;F_{4}\right\}=f_{Backbone}\left(X\right),$$

where $f_{Backbone}\left(\cdot \right)$ denotes the feature extraction process consisting of the stem, four stages, and three downsampling layers.

Conventional ConvNeXtV2-based classification models generally use only the final-stage feature, $F_{4}$, for classification. However, this approach may not fully exploit the low-level structural information and intermediate-level representations learned in the earlier stages. Therefore, the proposed method extracts hierarchical features from all four stages. These features are aligned within a common feature representation space using the Multi-Level FA module described in the following section and are subsequently used for adaptive multi-layer feature fusion.

### 3.3. Multi-Level Feature Alignment

The hierarchical features $F_{1},\;F_{2},\;F_{3},$ and $F_{4}$ generated by the four stages of ConvNeXtV2-B differ in spatial resolution, channel dimension, and semantic representation level. Because these features exist in different feature representation spaces, directly fusing them may lead to dimensional inconsistencies and feature mismatches, thereby limiting the effective utilization of complementary information across hierarchical levels. Therefore, before applying the AMFF module, the FA module aligns all hierarchical features within a common feature representation space.

First, an independently learnable 1 × 1 convolution is applied to the feature map from each stage to project its channel dimension into a common dimension, C. The channel projection process for the i-th stage feature is defined as follows:

(5) $${\tilde{F}}_{i}=P_{i}\left(F_{i}\right)={Conv}_{1\times 1}^{\left(i\right)}\left(F_{i}\right),\;\;i\in \left\{1,\;2,\;3,\;4\right\},$$

where $P_{i}\left(\cdot \right)$ denotes the channel projection function for the i-th stage, and ${Conv}_{1\times 1}^{\left(i\right)}\left(\cdot \right)$ represents a learnable 1 × 1 convolution that transforms the original channel dimension $C_{i}$ into the common dimension C. After channel projection, each feature has the following dimensions:

(6) $${\tilde{F}}_{i}\in R^{H_{i}\times W_{i}\times C},$$

The 1 × 1 convolution reorganizes the channel information from each stage into a common representation space without changing the spatial resolution. In addition, independent projection parameters are learned for each stage, enabling low-level structural features and high-level semantic features to be transformed according to their respective feature distributions, thereby improving semantic consistency across different hierarchical levels.

After the channel dimensions have been unified, the spatial resolutions of all features are aligned with the Stage 4 output resolution of H/32×W/32. The spatial alignment process is defined as follows:

(7) $${\hat{F}}_{i}=A_{i}\left({\tilde{F}}_{i}\right),\;\;i\in \left\{1,\;2,\;3,\;4\right\},$$

where $A_{i}\left(\cdot \right)$ denotes the spatial alignment operation applied to the i-th stage feature, and ${\hat{F}}_{i}$ represents the final aligned feature. The features from Stages 1, 2, and 3 are downsampled using the required scaling factors, whereas the Stage 4 feature is retained without additional spatial transformation because it already has the target resolution.

After channel and spatial alignment, all hierarchical features have identical dimensions:

(8) $${\hat{F}}_{i}\in R^{\frac{H}{32}\times \frac{W}{32}\times C},\;\;i\in \left\{1,\;2,\;3,\;4\right\},$$

Accordingly, the final aligned multi-level feature set is defined as follows:

(9) $$\hat{F}=\left\{{\hat{F}}_{1},\;{\hat{F}}_{2},\;{\hat{F}}_{3},\;{\hat{F}}_{4}\right\},$$

In this study, all features are aligned with the Stage 4 output, which has the lowest spatial resolution. Compared with repeatedly upsampling high-level features, this design reduces memory consumption and computational complexity while allowing features from different hierarchical levels to be represented within the same spatial coordinate system. Furthermore, transforming the detailed structural information extracted from the earlier stages into a representation that is spatially compatible with the semantic features from the later stages improves the stability and efficiency of the subsequent hierarchical feature fusion process.

The channel projection layers are jointly trained with the backbone network in an end-to-end manner using the final classification loss. The aligned feature set, $\hat{F}$, is subsequently passed to the AMFF module.

### 3.4. Adaptive Multi-Layer Feature Fusion

The aligned features ${\hat{F}}_{1},\;{\hat{F}}_{2},\;{\hat{F}}_{3},\;$ and ${\hat{F}}_{4}$ generated by the FA module have identical channel dimensions and spatial resolutions and can therefore be fused directly. Because lesion size, morphology, texture, and other visual characteristics vary across medical images, the relative contribution of hierarchical features may also vary across inputs. Fixed-weight fusion applies the same stage weights to all input images, whereas the proposed method computes the relative contribution of each stage separately for each input image. Accordingly, an input-dependent stage-level weighting strategy is employed to integrate the aligned hierarchical features.

To this end, the proposed method employs an Adaptive Multi-Layer Feature Fusion (AMFF) module. AMFF estimates the importance of each stage based on its global feature representation and adaptively fuses hierarchical features according to the characteristics of the input medical image. The resulting C-dimensional feature vector is used to compute a single scalar score for the corresponding stage, rather than separate channel-wise fusion weights.

First, GAP is applied to each aligned feature to extract a global feature representation. The global representation of the i-th stage feature is defined as follows:

(10) $$g_{i}=GAP\left({\hat{F}}_{i}\right),\;\;g_{i}\in R^{C},$$

where $g_{i}^{\left(c\right)}$ denotes the global response of the c-th channel in the i-th stage feature, and $g_{i}$ represents the feature vector composed of the global responses of all channels. GAP removes the spatial dimensions while summarizing the overall activation distribution of each stage feature.

An independently learnable linear transformation is then applied to each global feature vector to calculate the stage-importance score, $s_{i}$:

(11) $$s_{i}=W_{i}g_{i}+b_{i},\;\;i\in \left\{1,\;2,\;3,\;4\right\},$$

where $W_{i}\in R^{1\times C}\;$ and $b_{i}\in R$ are learnable parameters for estimating the importance of the i-th stage, and $s_{i}\in R$ denotes the corresponding unnormalized importance score. Applying an independent importance estimator to each stage enables features with different levels of abstraction to be evaluated according to their respective representation characteristics.

The four importance scores are subsequently normalized using the Softmax function. The normalized weight, $\alpha_{i}$, representing the relative contribution of the *i*-th stage, is calculated as follows:

(12) $$\alpha_{i}=\frac{exp\left(s_{i}\right)}{\sum_{j=1}^{4}exp\left(s_{j}\right)},\;\;i\in \left\{1,\;2,\;3,\;4\right\},$$

The normalized weights satisfy the following conditions:

(13) $$0{<\alpha }_{i}<1,\;\;\sum_{i=1}^{4}\alpha_{i}=1,$$

The Softmax function determines the relative contribution of each stage by jointly considering all stage-importance scores. Furthermore, $\alpha_{i}$ is not a fixed constant after training but an input-dependent weight dynamically computed from the features of each input image.

The final fused feature is generated by applying the normalized stage weights to the corresponding aligned features and combining them through element-wise summation:

(14) $$F_{AMFF}=\sum_{i=1}^{4}\alpha_{i}{\hat{F}}_{i},$$

Each $\alpha_{i}$ is applied uniformly across all channels and spatial locations of the corresponding stage feature. Because all features have been transformed into a common representation through the feature alignment process, the final fused feature has the following dimensions:

(15) $$F_{AMFF}\in R^{\frac{H}{32}\times \frac{W}{32}\times C},$$

Unlike concatenation-based fusion, AMFF does not increase the number of output channels. As shown in Equation (14), AMFF computes the relative contribution of each stage for each input image and combines the aligned hierarchical features through weighted element-wise summation. Consequently, low-level structural information from the earlier stages and high-level semantic information from the later stages are integrated using input-dependent stage-level weights. Each $\alpha_{i}$ is applied uniformly across all channels and spatial locations of the corresponding stage feature; therefore, AMFF performs dynamic adaptation at the stage level rather than at the channel or spatial level.

However, $\alpha_{i}$ is a scalar weight applied to an entire stage and therefore does not directly adjust the importance of individual channels within the fused feature. In addition, channel responses that are redundant or less relevant to classification may remain after feature fusion. To address this limitation, the ECA module is applied to $F_{AMFF}$ to model inter-channel interactions and recalibrate the fused feature at the channel level.

### 3.5. Efficient Channel Attention

The ECA module learns inter-channel interactions within the fused feature $F_{AMFF}$ to emphasize channel responses that are informative for classification while suppressing redundant or less relevant responses. Unlike conventional channel attention mechanisms that employ fully connected layers with channel dimensionality reduction, ECA uses a lightweight one-dimensional convolution to model local inter-channel relationships while introducing only a small number of additional parameters.

First, GAP is applied to $F_{AMFF}$ to generate the channel descriptor z, which consists of the global response of each channel:

(16) $$z=GAP\left(F_{AMFF}\right),\;\;z\in R^{C},$$

A one-dimensional convolution with kernel size k is then applied to the channel descriptor z to learn local interactions among neighboring channels, and a sigmoid activation function is used to generate the channel-wise importance weights, a:

(17) $$a=\sigma \left(Conv1D_{k}\left(z\right)\right),\;\;a=\left\{a_{1},\;a_{2},\;\ldots ,\;a_{C}\right\},\;\;a_{c}\in \left(0,\;1\right),$$

where $a_{c}$ represents the relative importance of the c-th channel. Because ECA does not reduce the channel dimension, it can model inter-channel relationships while avoiding the information loss associated with dimensionality reduction and reconstruction.

The resulting channel weights are applied to the corresponding channels of the fused feature:

(18) $$F_{ECA}=a⊙F_{AMFF},$$

where ⊙ denotes channel-wise element-wise multiplication. Each channel weight is uniformly applied across all spatial locations of the corresponding channel. Consequently, ECA selectively recalibrates channel responses without changing either the spatial resolution or number of channels.

Whereas AMFF adjusts feature importance at the stage level according to the input image, ECA further refines the importance of individual channels within the fused feature. This sequential feature refinement at both the hierarchical and channel levels enhances information that is beneficial for classification while suppressing redundant or less informative responses. The resulting feature, $F_{ECA}$, is subsequently passed to the classification head.

### 3.6. Classification Head and Objective Function

The channel-recalibrated feature $F_{ECA}$ generated by the ECA module is passed to the final classification head. The classification head consists of Global Average Pooling (GAP) followed by a fully connected layer, which transforms the refined feature representation into the class space to generate the final prediction for the input medical image.

First, GAP is applied to $F_{ECA}$ to remove the spatial dimensions and generate the channel-wise global feature vector f:

(19) $$f=GAP\left(F_{ECA}\right),\;\;f\in R^{C},$$

Compared with directly flattening the feature map, GAP reduces the number of parameters in the classification layer while preserving the channel information emphasized by the ECA module. The resulting global feature vector is transformed into the class-wise logits o through a fully connected layer:

(20) $$o=W_{cls}f+b_{cls},\;\;o\in R^{K},$$

where the classification parameters have the following dimensions:

(21) $$W_{cls}\in R^{K\times C},\;\;b_{cls}\in R^{K},$$

and K denotes the total number of classes. The resulting logits are processed using an output function and a classification loss appropriate for the label structure of each dataset. For single-label multiclass datasets, the Cross-Entropy Loss is used during training, and the Softmax function is applied to obtain class probabilities. For the multi-label ChestXray14 dataset, BCEWithLogitsLoss is applied directly to the logits during training, thereby combining the sigmoid activation and binary cross-entropy loss in a numerically stable manner.

To improve the reproducibility of the proposed architecture, the layer-wise feature representations and the main architectural parameters of AMFF-Net are summarized in Table 1. ConvNeXtV2-B provides hierarchical feature representations from four stages with progressively reduced spatial resolutions and increased channel dimensions. The FA module projects the features from each stage into a common 256-dimensional representation space and aligns their spatial resolutions to that of Stage 4. Specifically, the features from Stages 1, 2, and 3 are downsampled using stage-specific average pooling, while the Stage 4 feature is retained without additional spatial transformation. No manually specified stage-specific fusion weights are used. Instead, AMFF computes an input-dependent scalar weight for each stage. The aligned features are subsequently processed by AMFF using input-dependent stage-level scalar weights, which are uniformly applied to all channels and spatial locations within each stage feature. The resulting fused representation is further refined by ECA through lightweight channel-wise recalibration.

AMFF-Net is trained end-to-end by minimizing the final classification loss. All learnable parameters, including those of the ConvNeXtV2-B backbone, FA projection layers, AMFF stage-importance estimators, ECA module, and classification head, are jointly optimized. Thus, hierarchical feature extraction, feature alignment, input-dependent stage-level fusion, and channel-wise recalibration are jointly learned according to the final classification objective.

## 4. Experiments and Results

### 4.1. Datasets

To evaluate the proposed AMFF-Net across diverse medical imaging scenarios, three publicly available datasets—Kvasir-v2, HAM10000, and ChestXray14—were used. For Kvasir-v2 and HAM10000, stratified random splitting was performed once at a ratio of 70:10:20, and the resulting partitions were fixed across all models and three repeated runs. For ChestXray14, the official patient-wise split was consistently maintained across all models and repeated runs to prevent patient-level data leakage and ensure fair comparison [36]. All images were resized to 224 × 224 pixels.

Kvasir-v2 is a publicly available gastrointestinal endoscopy image dataset developed by the Simula Research Laboratory and Vestre Viken Health Trust. It contains 8000 RGB images across eight classes, including gastrointestinal lesions and normal anatomical structures, with 1000 images per class. Thus, the dataset is class-balanced and does not exhibit substantial class-level data imbalance. The original image resolutions range from 720 × 576 to 1920 × 1072 pixels.

HAM10000 (Human Against Machine with 10,000 Training Images) is a publicly available dataset for skin lesion classification, containing 10,015 dermoscopic images collected from multiple medical institutions and categorized into seven disease classes. Unlike Kvasir-v2, HAM10000 exhibits substantial class imbalance, with the majority class, melanocytic nevus, containing approximately 6705 images, compared with only 115 images in the minority class, dermatofibroma, resulting in a majority-to-minority class ratio of approximately 58:1. The dataset exhibits substantial variations in lesion size, shape, color, and acquisition conditions, making it suitable for evaluating model generalization under class-imbalanced distributions.

ChestXray14 is a large-scale chest X-ray dataset released by the National Institutes of Health (NIH), containing 112,120 images from 30,805 patients. It provides annotations for 14 thoracic diseases and is formulated as a multi-label classification problem because multiple diseases may be present in a single image. The prevalence of individual disease labels is highly uneven, with common findings having substantially more positive samples than rare findings such as hernia and pneumothorax. Thus, ChestXray14 also presents a substantial label-frequency imbalance inherent to the multi-label classification setting. Accordingly, model performance was evaluated using macro AUC to provide an overall measure of discriminative performance across the 14 disease categories while reducing the influence of label-frequency imbalance.

### 4.2. Experimental Setup

All experiments were implemented using PyTorch 2.2 on a workstation equipped with an Intel Core i9-14900K CPU, 64 GB RAM, and an NVIDIA GeForce RTX 4090 GPU with 24 GB memory. The experimental environment was configured with Ubuntu 22.04, CUDA 12.2, and cuDNN 9.0. AMFF-Net employed ConvNeXtV2-B initialized with the officially released ImageNet-pretrained weights. The baseline models, including ConvNeXt, ConvNeXtV2, EfficientNetV2, and ViT-B/16, were initialized with their corresponding official ImageNet-pretrained weights.

For each dataset, the training, validation, and test partitions were generated once and fixed throughout all experiments. Kvasir-v2 and HAM10000 were divided into stratified training, validation, and test sets at a ratio of 7:1:2, whereas the official patient-level partition of ChestXray14 was maintained. All compared models were trained and evaluated using exactly the same dataset partitions within each dataset, and these partitions remained unchanged across all three repeated runs. Different random seeds affected only stochastic training processes, such as model initialization and mini-batch ordering, and did not alter the data partitions. The test sets were strictly held out from model training, model selection, and hyperparameter tuning.

The main training and evaluation settings are summarized in Table 2.

During training, all compared models followed the same preprocessing pipeline, dataset-specific augmentation policy, optimization strategy, and evaluation protocol. Horizontal flipping was excluded for ChestXray14 to preserve anatomical laterality. Cross-Entropy Loss was used for Kvasir-v2, class-weighted Cross-Entropy Loss for HAM10000, and BCEWithLogitsLoss for ChestXray14.

### 4.3. Evaluation Metrics

To comprehensively evaluate AMFF-Net, accuracy, precision, recall, macro F1-score, and AUC were used as evaluation metrics. Accuracy measures the proportion of correctly classified samples, while precision and recall quantify the correctness of positive predictions and the ability to identify positive samples, respectively. Accuracy is defined as follows:

(22) $$Accuracy=\frac{TP+TN}{TP+TN+FP+FN},$$

where TP, TN, FP, and FN represent true positive, true negative, false positive, and false negative, respectively.

Precision and recall are calculated as follows:

(23) $$Precision=\frac{TP}{TP+FP}\;\;,\;\;\;\;Recall=\frac{TP}{TP+FN},$$

The F1-score for each class is defined as the harmonic mean of precision and recall, while the macro F1-score is calculated as the equally weighted average of the class-wise F1-scores to account for class imbalance.

(24) $$F1=\frac{2PR}{P+R},$$

where P and R denote precision and recall, respectively.

Because ChestXray14 is a multi-label classification problem, accuracy alone may not fully reflect model performance. Therefore, AUC was additionally used to assess the discriminative ability of the models across different decision thresholds. The receiver operating characteristic (ROC) curve represents the relationship between the true positive rate (TPR) and false positive rate (FPR), and AUC is defined as the area under the ROC curve:

(25) $$TPR=\frac{TP}{TP+FN}\;\;,\;\;\;\;FPR=\frac{FP}{FP+TN},$$

(26) $$AUC=\int_{0}^{1}TPR\left(FPR\right)d(FPR),$$

(27) $$Macro\;AUC=\frac{1}{C}\sum_{c=1}^{C}{AUC}_{c},$$

An AUC closer to 1 indicates stronger discrimination, whereas 0.5 represents random classification. Since ChestXray14 is a multi-label dataset, AUC was calculated for each disease class and equally averaged to obtain the macro AUC, as shown in Equation (27), where C denotes the total number of disease classes and ${AUC}_{c}$ denotes the AUC for the *c*-th class.

All comparison models were evaluated on the same test dataset. Each experiment was repeated three times with different random seeds, and the mean and standard deviation were reported to improve reproducibility and statistical reliability.

### 4.4. Comparison with Representative Baseline Methods

To objectively evaluate the performance of the proposed AMFF-Net, it was compared with ConvNeXt (ConvN), ConvNeXtV2 (ConvV2), EfficientNetV2 (EN2), and Vision Transformer (ViT). For a fair comparison, all models were evaluated using the same fixed data partitions, input resolution, dataset-specific data augmentation strategy, optimization algorithm, and learning-rate schedule. Accuracy, precision, recall, and F1-score were used as the evaluation metrics for the Kvasir-v2 and HAM10000 datasets. For ChestXray14, a multi-label classification dataset with substantial variation in disease prevalence across disease categories, label-wise accuracy and macro AUC were used as the evaluation metrics. The macro AUC was calculated by first obtaining the AUC for each disease category and then averaging the class-wise AUC values with equal weighting, thereby providing a balanced evaluation across disease categories with different prevalence.

Table 3 presents the performance of each model on the Kvasir-v2 single-label multiclass classification task. AMFF-Net achieved the best performance across all evaluation metrics, with an accuracy of 96.72%, a macro precision of 96.79%, a macro recall of 96.18%, and a macro F1-score of 96.47%. Compared with ConvV2, which employs the same ConvNeXtV2-B backbone, AMFF-Net improved accuracy, precision, recall, and F1-score by 2.01, 1.87, 1.81, and 1.83 percentage points, respectively. In terms of accuracy, AMFF-Net also outperformed ConvN, EN2, and ViT by 3.77, 4.29, and 3.11 percentage points, respectively.

The Kvasir-v2 dataset contains normal anatomical structures, pathological findings, and images acquired during endoscopic procedures. Although some classes exhibit similar overall structures, they differ in mucosal color, surface texture, lesion boundaries, and local morphology. Therefore, representing both fine-grained local features and higher-level structural information is important for accurate classification.

AMFF-Net first aligns features extracted from different stages into a common representation space, thereby facilitating the integration of earlier-stage structural information with later-stage semantic representations. It then adaptively adjusts the contribution of each stage according to the input image, while the ECA module further recalibrates the fused representation at the channel level. These design characteristics provide a plausible explanation for the consistent improvements observed across accuracy, precision, recall, and F1-score.

Furthermore, the standard deviation of the accuracy achieved by AMFF-Net was 0.43 percentage points, compared with 0.71 percentage points for ConvV2. This result suggests that AMFF-Net exhibited more consistent classification performance across repeated training runs with different random seeds, despite variations arising from model initialization and stochastic optimization.

**Figure 3:**
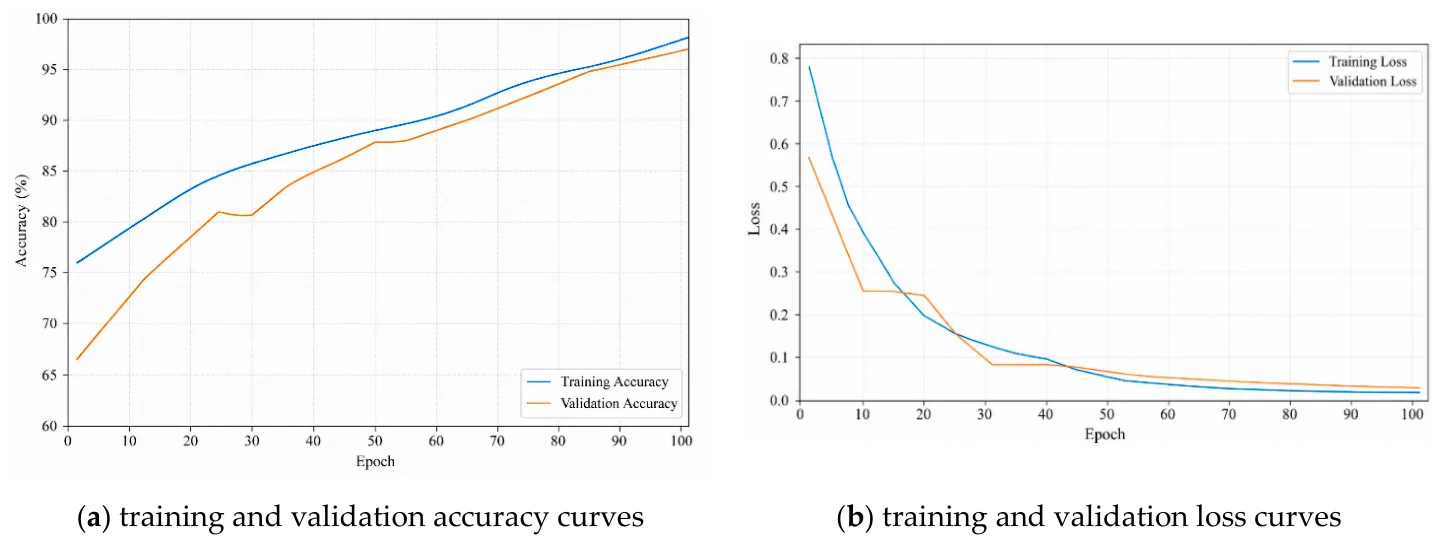
Training and validation performance of the proposed AMFF-Net on the Kvasir-v2 dataset over 100 epochs.

To further verify the convergence and training stability of the proposed AMFF-Net, the training and validation accuracy and loss curves over 100 epochs on the Kvasir-v2 dataset are presented in Figure 3. As shown in Figure 3a, both training and validation accuracy progressively increase and stabilize during the later epochs. Figure 3b shows that both training and validation losses consistently decrease and converge to low values without a sustained increase in validation loss, indicating stable training and no severe overfitting. The relatively small gap between the training and validation performance further suggests reasonable generalization. These results provide additional support for the reliability of the proposed architecture, while the final accuracy of 96.72% reported in Table 3 corresponds to the independent test-set evaluation.

Table 4 presents the performance of each model on the HAM10000 single-label multiclass classification task. AMFF-Net achieved the best performance across all evaluation metrics, with an accuracy of 95.25%, a macro precision of 91.35%, a macro recall of 91.10%, and a macro F1-score of 91.22%. Compared with ConvV2, AMFF-Net improved accuracy by 2.10 percentage points, whereas precision, recall, and F1-score increased by 1.74, 1.87, and 1.80 percentage points, respectively. Its F1-score was also 2.82, 3.73, and 3.44 percentage points higher than those of ViT, ConvN, and EN2, respectively.

HAM10000 has a highly imbalanced class distribution, and lesions within the same class exhibit considerable variation in color, size, texture, and morphology. In addition, lesions from different classes often share similar visual characteristics. Therefore, macro-averaged metrics, which assign equal weight to each class, are particularly important for assessing performance across minority and majority classes in addition to overall accuracy. The improvements observed in macro precision, macro recall, and macro F1-score, together with the increase in accuracy, indicate that AMFF-Net achieved consistently stronger classification performance across the evaluated lesion categories.

AMFF-Net integrates representations from earlier stages that preserve fine-grained structural information, such as surface textures and lesion boundaries, with higher-level representations from deeper stages that capture lesion morphology and semantic information. The input-dependent stage-level fusion mechanism adjusts the relative contribution of each feature stage according to the characteristics of the input image, whereas the ECA module further refines the fused representation through channel-wise recalibration. These architectural characteristics provide a plausible explanation for the observed improvements in precision, recall, and F1-score, particularly for visually similar skin lesions.

Table 5 presents the multi-label classification performance of each model on the ChestXray14 dataset. Multiple diseases may coexist in a single ChestXray14 image, and the number of positive samples varies substantially across disease classes. Under these conditions, precision, recall, and F1-score can vary considerably depending on the decision threshold and the proportion of positive samples for each disease. Accordingly, previous studies using ChestXray14 have primarily employed disease-wise ROC-AUC and its macro average as the principal evaluation metrics [9,37,38,39].

Accordingly, Table 5 reports label-wise accuracy, which represents the binary prediction agreement across all image–disease label pairs, and macro AUC, which assigns equal weight to the ROC-AUC of each of the 14 disease classes. Label-wise accuracy was included as a supplementary metric and was calculated by applying the disease-specific thresholds determined on the validation set to the test set. In contrast, macro AUC was used as the primary evaluation metric because it evaluates the ranking ability of the model to distinguish positive and negative cases for each disease across different decision thresholds, without requiring a single fixed operating threshold.

Among the comparison models, ConvV2 achieved the strongest baseline performance, with a label-wise accuracy of 91.52% and a macro AUC of 87.58%. AMFF-Net achieved 94.15% and 89.86%, respectively, outperforming ConvV2 by 2.63 and 2.28 percentage points. Compared with ConvN, AMFF-Net improved these two metrics by 3.84 and 4.02 percentage points, respectively. The corresponding improvements over EN2 were 3.39 and 3.75 percentage points, whereas those over ViT were 3.08 and 2.91 percentage points.

Because ChestXray14 contains a high proportion of negative labels and substantial variation in disease prevalence across classes, accuracy alone may not fully reflect the model’s ability to discriminate between positive and negative disease labels, particularly across imbalanced disease classes. AMFF-Net achieved the highest performance not only in label-wise accuracy but also in macro AUC. These results indicate improved overall label agreement and stronger class-wise discrimination across the 14 disease categories.

Chest X-ray images contain disease-related features at multiple spatial scales, ranging from small local opacities and subtle boundary changes to extensive structural abnormalities involving the lungs, heart, and thoracic cavity. AMFF-Net integrates representations from earlier stages that preserve fine-grained structural information with higher-level anatomical and semantic representations from deeper stages through input-dependent stage-level adaptive fusion. The ECA module further refines the fused representation through channel-wise recalibration. These architectural characteristics provide a plausible explanation for the observed improvements in both label-wise accuracy and macro AUC.

Overall, AMFF-Net achieved the best performance among the evaluated models across the three datasets: Kvasir-v2, in which mucosal color and texture are important; HAM10000, which is characterized by class imbalance and high visual similarity among lesion classes; and ChestXray14, which involves disease-related features at multiple spatial scales. In particular, AMFF-Net consistently outperformed ConvV2, which employs the same ConvNeXtV2-B backbone, supporting the effectiveness of the proposed integrated framework for hierarchical feature utilization.

### 4.5. Ablation Study

An ablation study was conducted on the Kvasir-v2 dataset to evaluate the contribution of each component of AMFF-Net to the overall classification performance. The same data split, training conditions, and hyperparameters were used for all model variants to ensure a consistent comparison. A cumulative evaluation strategy was adopted, in which the proposed modules were sequentially incorporated into the ConvNeXtV2-B baseline. The evaluated configurations consisted of (1) the ConvNeXtV2-B baseline, (2) the baseline with the FA module, (3) the model incorporating both the FA and AMFF modules, and (4) the complete AMFF-Net comprising the FA, AMFF, and ECA modules.

Table 6 presents the changes in classification performance as the proposed modules were sequentially incorporated. The ConvNeXtV2-B baseline achieved an accuracy of 94.71%, a precision of 94.92%, a recall of 94.37%, and an F1-score of 94.64%. After adding the FA module, the accuracy increased by 0.66 percentage points to 95.37%. Precision, recall, and F1-score also improved by 0.56, 0.73, and 0.64 percentage points, respectively. These results indicate that aligning features from different network stages into a common representation space can provide a useful basis for integrating hierarchical feature representations.

When the AMFF module was added to the FA-based model, the accuracy increased to 96.16%, representing an improvement of 0.79 percentage points over the FA-only model and 1.45 percentage points over the baseline. Precision, recall, and F1-score also improved by 0.81, 0.64, and 0.73 percentage points, respectively, relative to the FA-only model. These results support the effectiveness of incorporating the AMFF module into the aligned multi-stage feature representation, although this cumulative ablation alone does not isolate the independent effect of input-dependent stage-level weighting from that of multi-stage feature fusion itself.

The complete AMFF-Net, incorporating the ECA module in addition to FA and AMFF, achieved the best performance, with an accuracy of 96.72%, a precision of 96.79%, a recall of 96.18%, and an F1-score of 96.47%. Compared with the model incorporating only FA and AMFF, the complete model further improved accuracy by 0.56 percentage points, precision by 0.50 percentage points, recall by 0.44 percentage points, and F1-score by 0.46 percentage points. These improvements support the role of ECA as an additional refinement mechanism for the fused representation through channel-wise recalibration.

Compared with the ConvNeXtV2-B baseline, the complete AMFF-Net improved accuracy, precision, recall, and F1-score by 2.01, 1.87, 1.81, and 1.83 percentage points, respectively. Overall, the progressive improvement observed as FA, AMFF, and ECA were sequentially incorporated supports the complementary contributions of these components within the integrated AMFF-Net framework. Specifically, FA establishes a common representation space for multi-stage features, AMFF adaptively integrates the aligned stage-level representations, and ECA further refines the resulting fused representation through channel-wise recalibration. Accordingly, the ablation results provide evidence for the effectiveness of the proposed integrated framework and the contribution of the AMFF module as implemented, but do not establish the independent superiority of input-dependent stage-level weighting over fixed or non-adaptive fusion strategies.

### 4.6. Computational Complexity Analysis

The practical applicability of a medical image classification model depends not only on its classification performance but also on its computational cost and resource requirements. Accordingly, the performance–cost trade-off of AMFF-Net was evaluated by comparing classification accuracy, parameter count, GFLOPs, GPU memory usage, and average inference time per image.

All models were evaluated under the same experimental conditions using an input resolution of 224 × 224 pixels and a batch size of 1 on an NVIDIA GeForce RTX 4090 GPU. Inference time was measured for the model forward pass only, excluding image preprocessing and model-loading time. Before measurement, 10 warm-up iterations were performed to minimize initialization and GPU runtime effects. Subsequently, 100 forward passes were measured, with GPU synchronization performed immediately before and after each timed forward pass to account for the asynchronous execution of CUDA operations. The average execution time across the 100 measurements was reported as the inference time per image. The same measurement protocol was applied to all compared models.

Table 7 summarizes the classification performance and computational characteristics of the evaluated models on the Kvasir-v2 dataset. AMFF-Net achieved the highest classification accuracy (96.72%). Compared with ConvV2, which employs the same ConvNeXtV2-B backbone, AMFF-Net improved accuracy by 2.01 percentage points, whereas the number of parameters increased from 87.52 M to 88.81 M, corresponding to an increase of 1.29 M (1.47%). The computational cost increased from 15.35 GFLOPs to 16.12 GFLOPs, representing an increase of 0.77 GFLOPs (5.02%). These results indicate that the proposed FA, AMFF, and ECA modules introduce a relatively modest additional computational cost while providing a clear improvement in classification performance.

The average inference time of AMFF-Net was 13.02 ms per image, which was 0.76 ms longer than the 12.26 ms recorded for ConvV2. This relatively small increase in inference latency suggests that the additional feature alignment and adaptive fusion operations introduce limited inference overhead under the evaluated GPU setting. Under the same experimental conditions, this inference speed corresponds to approximately 77 images per second.

GPU memory usage increased by 116.87 MB (8.62%), from 1355.48 MB for ConvV2 to 1472.35 MB for AMFF-Net. This increase reflects the additional memory required to retain intermediate-stage feature maps and perform hierarchical feature fusion. Nevertheless, AMFF-Net required 101.45 MB less GPU memory than ViT despite its multi-stage feature processing.

EN2 exhibited the lowest computational complexity, requiring 52.46 M parameters, 5.22 GFLOPs, and 1188.66 MB of GPU memory. It also achieved the shortest inference time of 9.36 ms per image. However, its classification accuracy was 4.29 percentage points lower than that of AMFF-Net. These results illustrate a clear performance–cost trade-off: EN2 provides lower computational cost and faster inference, whereas AMFF-Net achieves substantially higher classification accuracy at the expense of additional computational resources.

ViT exhibited the highest theoretical computational cost and GPU memory usage, requiring 16.85 GFLOPs and 1573.80 MB, respectively. Its inference time was 13.87 ms per image, which was 0.85 ms longer than that of AMFF-Net. Compared with ViT, AMFF-Net achieved 3.11 percentage points higher classification accuracy while requiring 0.73 fewer GFLOPs and 101.45 MB less GPU memory. Under the same experimental conditions, these results indicate that AMFF-Net provides higher classification accuracy with lower computational cost, GPU memory usage, and inference latency than ViT.

Compared with ConvNeXt, AMFF-Net required an additional 1.29 M parameters and 0.77 GFLOPs, while improving classification accuracy by 3.77 percentage points. The difference in inference time was 1.04 ms. These findings indicate that AMFF-Net achieves a clear improvement in classification performance with relatively modest additional computational cost compared with the ConvNeXt baseline.

Overall, on the Kvasir-v2 dataset, AMFF-Net improved classification accuracy over ConvV2 by 2.01 percentage points, with increases of 1.47% in parameter count, 5.02% in GFLOPs, 8.62% in GPU memory usage, and approximately 0.76 ms in inference time per image. These results indicate a favorable performance–cost trade-off, in which the proposed architecture achieves a substantial classification improvement with relatively modest additional computational and memory requirements. However, AMFF-Net does not provide lower computational cost than ConvV2 and therefore should not be interpreted as being absolutely more computationally efficient than the baseline. Since the detailed computational analysis was conducted on Kvasir-v2, this performance–cost assessment is specifically supported for the evaluated Kvasir-v2 setting and should not be generalized to the other datasets without additional measurements.

### 4.7. Visualization and Qualitative Analysis

To qualitatively examine the image regions associated with model predictions, Gradient-weighted Class Activation Mapping (Grad-CAM) was applied to two polyp images from Kvasir-v2, two melanoma images from HAM10000, and two cardiomegaly images from ChestXray14 [40]. Using the same input images and Grad-CAM protocol, the spatial distributions of the highlighted regions were compared among EfficientNetV2 (EN2), ViT, ConvNeXtV2 (ConvV2), and AMFF-Net. In the visualizations, red indicates relatively strong contributions to the target-class prediction, whereas green and blue indicate relatively weak contributions.

**Figure 4:**
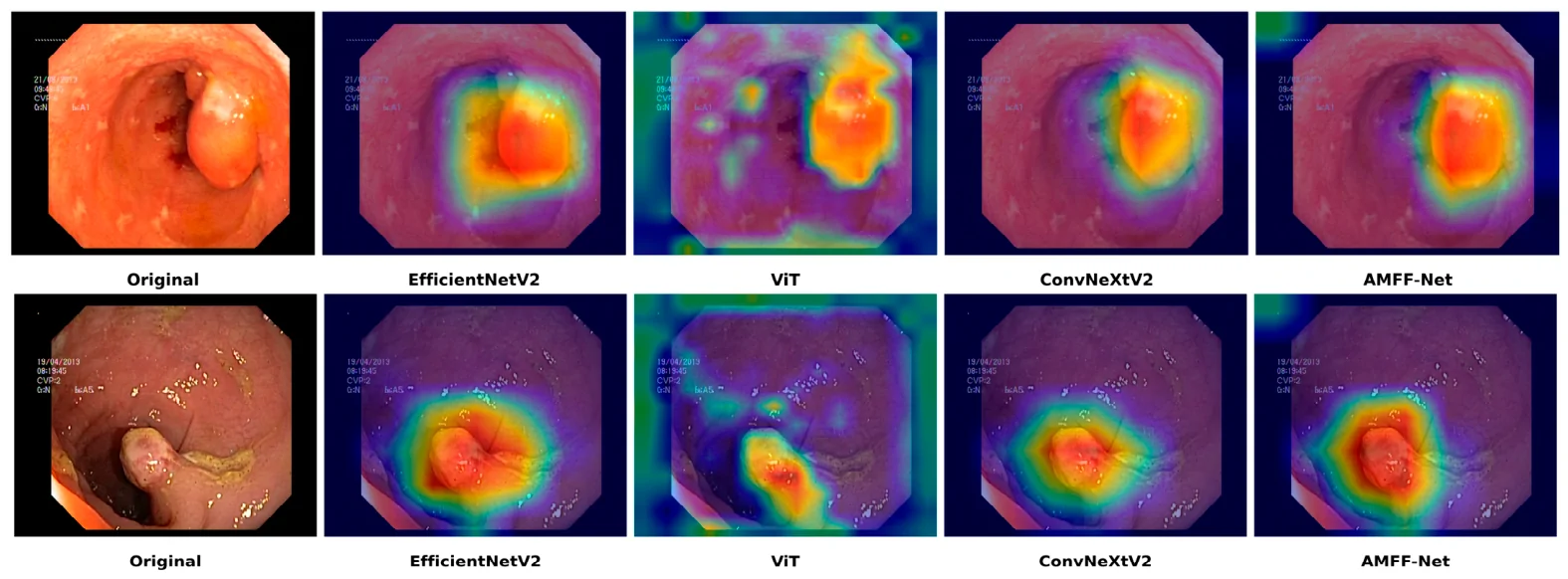
Qualitative comparison of Grad-CAM visualizations generated by different models on representative samples from the Kvasir-v2 dataset.

Figure 4 presents the Grad-CAM results for two polyp cases from Kvasir-v2. Since polyps are characterized by their protruding morphology, boundaries, color differences from the surrounding mucosa, and surface and vascular patterns [41,42], the analysis examined whether each model focused on the lesion and its boundary while suppressing irrelevant background regions. In the first case, EfficientNetV2 highlighted the lower portion and boundary of the polyp, while ViT showed fragmented activation extending beyond the lesion. ConvNeXtV2 exhibited strong but localized activation within the polyp, whereas AMFF-Net produced broader and stronger activation across the lesion and along its boundary. In the second case, ViT again showed activation in unrelated peripheral regions, while the other models highlighted the lesion, with AMFF-Net showing the most concentrated activation within the polyp. Overall, AMFF-Net consistently produced continuous and lesion-centered activation patterns, supporting its hierarchical feature fusion strategy that integrates low-level boundary and texture information with higher-level morphological and semantic representations, potentially contributing to the improved classification performance on Kvasir-v2 (Table 3).

**Figure 5:**
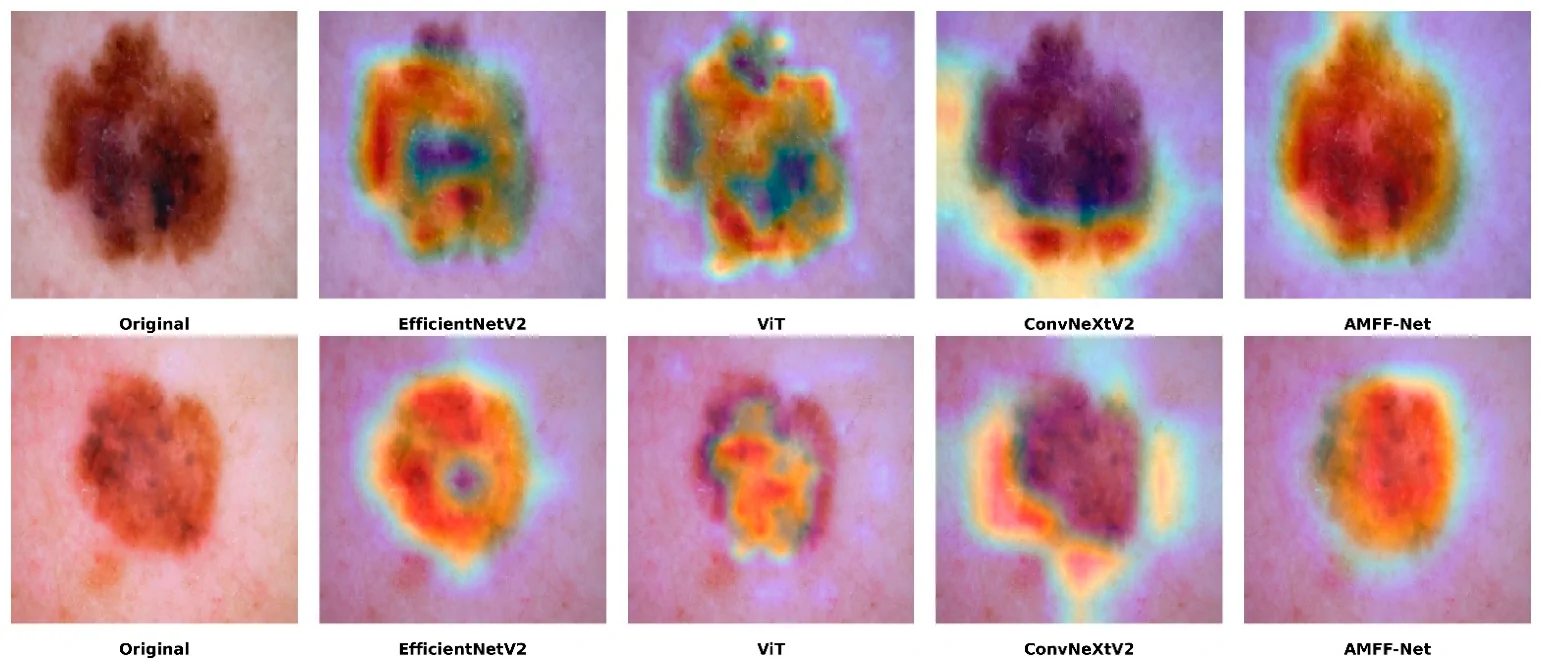
Qualitative comparison of Grad-CAM visualizations generated by different models on representative samples from the HAM10000 dataset.

Figure 5 presents the Grad-CAM results for two melanoma cases from HAM10000. Given the importance of irregular boundaries, heterogeneous pigmentation, and texture variation in melanoma assessment [43,44], the analysis focused on whether each model highlighted these visually relevant lesion regions. Across both cases, EfficientNetV2 and ViT showed relatively localized or fragmented activations, while ConvNeXtV2 primarily focused on localized boundary regions, with some activation extending into the surrounding skin. In contrast, AMFF-Net produced broader and more continuous activation across regions exhibiting pigmentation and texture variations and toward the lesion boundaries. These qualitative patterns are consistent with the proposed hierarchical feature fusion strategy, which integrates low-level structural information with higher-level morphological and semantic representations, and may help explain the improved classification performance on HAM10000 (Table 4).

**Figure 6:**
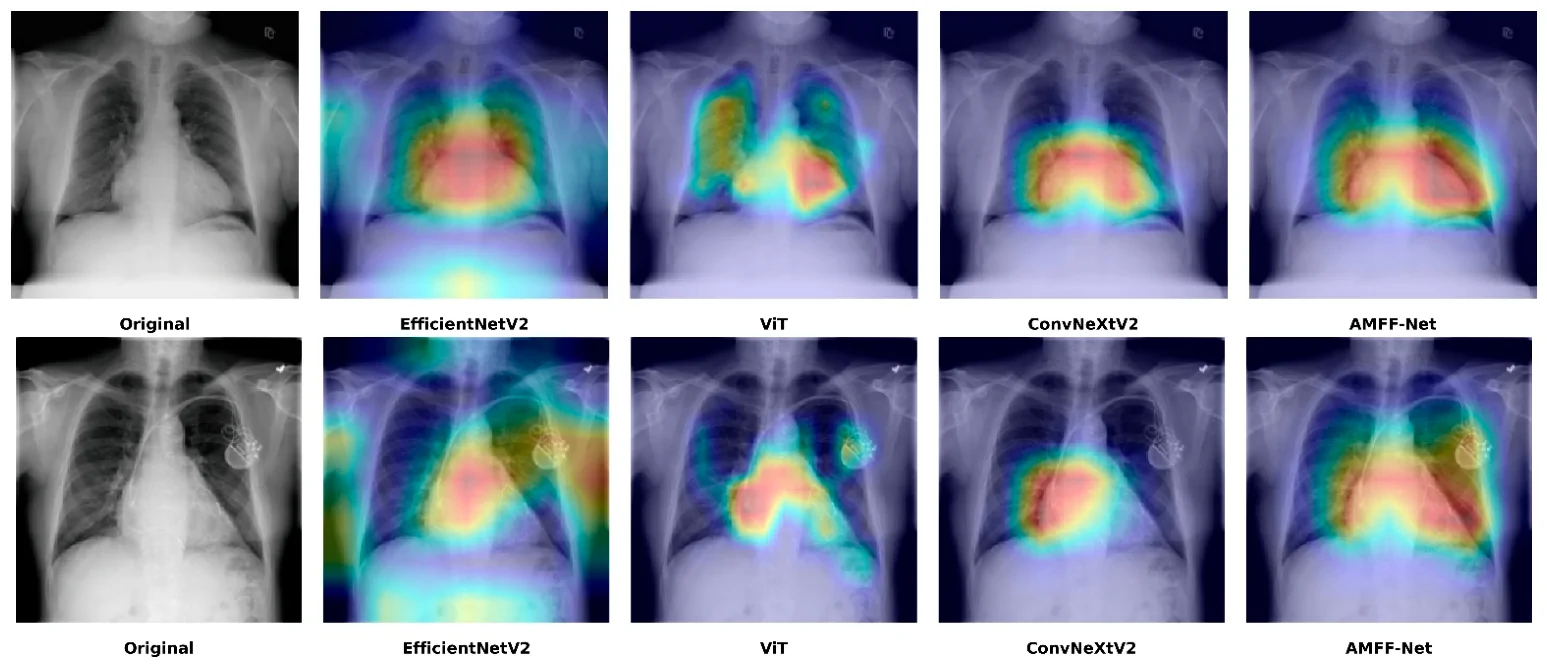
Qualitative comparison of Grad-CAM visualizations generated by different models for representative cardiomegaly cases from the ChestXray14 dataset.

Figure 6 presents the Grad-CAM results for two cardiomegaly cases from the ChestXray14 dataset. Cardiomegaly is commonly assessed on frontal chest radiographs using the cardiothoracic ratio (CTR), with a CTR greater than 0.5 generally considered suggestive of cardiac enlargement [45]. Accordingly, the analysis focused on the central cardiac silhouette, bilateral cardiac borders, and inferior cardiac contour.

In the first case, EfficientNetV2 showed strong activation in the central and inferior cardiac regions but also extended into the lung fields, while ViT produced relatively localized and fragmented activations. ConvNeXtV2 primarily highlighted the central cardiac region with weaker responses along the cardiac borders. In contrast, AMFF-Net produced broader and more continuous activation from the central cardiac region toward both borders and the inferior contour.

In the second case, EfficientNetV2 again showed activation extending beyond the cardiac region, whereas ViT and ConvNeXtV2 primarily highlighted localized central or left-sided regions with weaker responses along the enlarged right border and inferior contour. AMFF-Net produced broader and more continuous activation across the central and inferior cardiac regions and toward both cardiac borders.

Across both cases, AMFF-Net more consistently highlighted the central cardiac region, bilateral borders, and inferior contour than the comparison models. These qualitative patterns are consistent with the proposed hierarchical feature fusion strategy, which integrates local structural information with higher-level anatomical representations, and may help explain the improved macro AUC achieved on ChestXray14.

Overall, AMFF-Net produced relatively continuous and lesion-centered activation patterns within the polyp regions in Kvasir-v2, highlighted internal pigmentation and texture variations together with lesion boundaries in HAM10000, and produced broader activation across the central cardiac region, bilateral borders, and inferior contour in ChestXray14. Compared with the other models, AMFF-Net showed more spatially continuous activation patterns that qualitatively captured both local discriminative features and broader structural information. These observations, together with the quantitative results, provide complementary evidence supporting the effectiveness of the proposed adaptive multi-layer feature fusion strategy.

## 5. Discussion and Limitations

In this study, we proposed AMFF-Net, a ConvNeXtV2-B-based framework that exploits complementary representations from different network stages for medical image classification. Rather than relying solely on final-stage semantic features, AMFF-Net incorporates earlier-stage features that preserve fine-grained structural information.

Through FA and AMFF, multi-stage features are projected into a common representation space and fused using a single input-dependent scalar weight for each stage, allowing their relative contributions to adapt to each input image. The ECA module then performs lightweight channel recalibration on the fused representation. Thus, AMFF-Net integrates hierarchical feature fusion with channel recalibration, while AMFF specifically provides input-dependent stage-level adaptive fusion.

The consistent performance improvements observed on Kvasir-v2, HAM10000, and ChestXray14 suggest that AMFF-Net can effectively exploit hierarchical feature representations across the evaluated medical image classification settings, combining complementary low-level structural and high-level semantic information.

However, the current ablation study evaluates AMFF within the overall framework and does not independently isolate the effect of input-dependent stage-level weighting from the general benefit of multi-stage feature fusion. Because non-adaptive alternatives were not compared, the observed improvement supports the effectiveness of AMFF as implemented but does not establish the superiority of input-dependent stage-level weighting over input-independent fusion strategies. Such comparisons remain an important direction for future research.

Several additional limitations should be considered before practical clinical deployment.

First, the evaluation was limited to selected representative baseline architectures, including ConvNeXt, ConvNeXtV2, EfficientNetV2, and Vision Transformer. Comparisons with medical image classification methods based on ResNet, EfficientNet, or other task-specific architectures were not included. Direct numerical comparisons with previously reported methods were also not performed because differences in data splits, preprocessing, augmentation, training strategies, pretrained weights, and evaluation protocols may affect performance and limit fair cross-study comparisons. Therefore, the results should be interpreted as evidence of the effectiveness of AMFF-Net relative to the selected representative baselines, rather than as evidence of superiority over existing medical image classification methods. More systematic comparisons under standardized experimental conditions remain an important direction for future work.

Second, the proposed method was evaluated on publicly available datasets, including Kvasir-v2, HAM10000, and ChestXray14. Although these datasets cover diverse medical imaging settings, they may not fully reflect real-world clinical variability, such as inter-institutional domain shifts, differences in imaging devices and acquisition protocols, and variations in patient populations. Therefore, the present results do not establish cross-institutional generalization or robustness in clinical deployment. External validation using multi-center clinical datasets is required to further assess the generalization capability of AMFF-Net.

Third, AMFF-Net improves classification performance at the cost of additional computational and memory overhead compared with the ConvNeXtV2-B baseline, which may limit its use on resource-constrained devices or in real-time applications. Model compression techniques, such as knowledge distillation, pruning, and quantization, could be explored to reduce this overhead while maintaining classification performance.

Fourth, although Grad-CAM provides qualitative information about image regions associated with model predictions, it does not fully characterize the model’s decision-making process. Future studies should therefore incorporate quantitative explainability measures and complementary XAI methods to further assess the clinical relevance of the identified regions.

Fifth, although the evaluated datasets include varying degrees of class imbalance, the robustness of AMFF-Net under different imbalance conditions was not systematically evaluated.

Finally, the current framework uses image-based information alone; future studies could investigate its extension to multimodal medical AI by incorporating complementary clinical information.

## 6. Conclusions and Future Work

In this study, we proposed AMFF-Net, a ConvNeXtV2-B-based framework that integrates hierarchical multi-stage features through FA and AMFF, followed by lightweight channel recalibration using ECA. AMFF performs input-dependent stage-level adaptive fusion by assigning a single scalar weight to each network stage, thereby adapting the relative contribution of each stage to the input image.

Experimental results on Kvasir-v2, HAM10000, and ChestXray14 demonstrated consistent performance improvements in AMFF-Net over the ConvNeXtV2-B baseline and competitive performance against the selected representative CNN- and Transformer-based architectures. The ablation study showed progressive performance improvements with the sequential incorporation of FA, AMFF, and ECA. Computational analysis characterized the performance–cost trade-off of AMFF-Net, whereas Grad-CAM visualization provided qualitative evidence of feature localization. However, the present results do not establish the independent superiority of input-dependent stage-level weighting over fixed or non-adaptive fusion strategies, nor do they establish cross-institutional generalization or clinical deployment robustness.

Future work will focus on several directions to further validate and extend AMFF-Net. First, systematic comparisons with fixed and non-adaptive fusion strategies, such as feature summation, uniform averaging, and input-independent learnable stage weights, will be conducted to more explicitly assess the contribution of input-dependent stage-level weighting. Second, comparisons with additional medical image classification methods, including ResNet- and EfficientNet-based approaches, will be conducted under standardized experimental conditions. Third, external validation using multi-center clinical datasets will be performed to evaluate cross-institutional generalization under real-world conditions. Finally, the framework could be extended to multimodal medical AI by integrating complementary clinical information and to other medical image analysis tasks, such as detection and segmentation.

## Figures and Tables

**Table 1:** Layer-wise feature configurations and architectural parameters of AMFF-Net.

Component	Input/Feature	Spatial Resolution	Channels	Operation/Parameters
ConvNeXtV2-B Stage 1	Hierarchical feature	*H*/4 × *W*/4	128	Backbone feature extraction
ConvNeXtV2-B Stage 2	Hierarchical feature	*H*/8 × *W*/8	256	Backbone feature extraction
ConvNeXtV2-B Stage 3	Hierarchical feature	*H*/16 × *W*/16	512	Backbone feature extraction
ConvNeXtV2-B Stage 4	High-level feature	*H*/32 × *W*/32	1024	Backbone feature extraction
FA—Stage 1	Stage 1 feature	*H*/32 × *W*/32	256	1 × 1 convolution + average pooling (×8)
FA—Stage 2	Stage 2 feature	*H*/32 × *W*/32	256	1 × 1 convolution + average pooling (×4)
FA—Stage 3	Stage 3 feature	*H*/32 × *W*/32	256	1 × 1 convolution + average pooling (×2)
FA—Stage 4	Stage 4 feature	*H*/32 × *W*/32	256	1 × 1 convolution; no spatial downsampling
AMFF	Aligned multi-stage features	*H*/32 × *W*/32	256 per stage	Input-dependent stage-level scalar weighting
ECA	Fused feature	*H*/32 × *W*/32	256	Channel-wise recalibration
Classification Head	Refined feature	-	256 → K classes	Global pooling + linear classifier

Note: ***H*** and ***W*** denote the height and width of the input image, respectively. No manually specified stage-specific fusion weights are used.

**Table 2:** Training and evaluation settings used for AMFF-Net and the baseline models.

Category	Setting	Configuration
Input	Input resolution	224 × 224
	Normalization	ImageNet mean and standard deviation
Data split	Kvasir-v2/HAM10000	Stratified train/validation/test = 70/10/20
	ChestXray14	Fixed patient-level separation
	Data partition	Fixed and identical across all compared models
	Repeated runs	3
	Random seeds	Different seeds across runs
Optimization	Optimizer	AdamW
	Initial learning rate	1 × 10^−4^
	Weight decay	0.01
	Batch size	32
	LR warm-up	Linear, 5 epochs
	LR scheduler	Cosine annealing
	Minimum LR	1 × 10^−6^
	Maximum epochs	100
	Early stopping	Patience = 20
	Checkpoint	Lowest validation loss
Augmentation	Random horizontal flip	*p* = 0.5 *
	Random resized crop	224 × 224
	RandAugment	N = 2, M = 9
	Random erasing	*p* = 0.25
Training	Mixed precision	AMP

* Horizontal flipping was excluded for ChestXray14; the predefined dataset-specific augmentation policy was applied identically to all models within each dataset.

**Table 3:** Performance comparison on the Kvasir-v2 Dataset.

Model	Accuracy (%)	Precision (%)	Recall (%)	F1-Score (%)
ConvN	92.95 ± 0.63	93.18 ± 0.57	92.85 ± 0.62	93.01 ± 0.54
ConvV2	94.71 ± 0.71	94.92 ± 0.63	94.37 ± 0.46	94.64 ± 0.43
EN2	92.43 ± 0.56	92.57 ± 0.39	92.19 ± 0.69	92.39 ± 0.68
ViT	93.61 ± 0.49	93.85 ± 0.42	93.45 ± 0.54	93.64 ± 0.48
AMFF-Net	96.72 ± 0.43	96.79 ± 0.32	96.18 ± 0.35	96.47 ± 0.52

**Table 4:** Performance comparison on the HAM10000 Dataset.

Model	Accuracy (%)	Precision (%)	Recall (%)	F1-Score (%)
ConvN	91.35 ± 0.47	87.67 ± 0.78	87.31 ± 0.62	87.49 ± 0.57
ConvV2	93.15 ± 0.45	89.61 ± 0.52	89.23 ± 0.65	89.42 ± 0.55
EN2	91.75 ± 0.62	87.84 ± 0.80	87.73 ± 0.72	87.78 ± 0.73
ViT	92.19 ± 0.64	88.55 ± 0.59	88.26 ± 0.63	88.40 ± 0.61
AMFF-Net	95.25 ± 0.53	91.35 ± 0.65	91.10 ± 0.70	91.22 ± 0.68

**Table 5:** Performance comparison on the ChestXray14 Dataset.

Model	Label-Wise Accuracy (%)	Macro AUC (%)
ConvN	90.31 ± 0.57	85.84 ± 0.72
ConvV2	91.52 ± 0.54	87.58 ± 0.58
EN2	90.76 ± 0.61	86.11 ± 0.64
ViT	91.07 ± 0.49	86.95 ± 0.51
AMFF-Net	94.15 ± 0.64	89.86 ± 0.62

**Table 6:** Ablation study of the proposed AMFF-Net on the Kvasir-v2 Dataset.

Metric	Baseline	+FA	+FA + AMFF	AMFF-Net (FA + AMFF + ECA)
Feature Alignment	X	V	V	V
Adaptive Multi-Layer Feature Fusion	X	X	V	V
Efficient Channel Attention	X	X	X	V
Accuracy (%)	94.71	95.37	96.16	96.72
ΔAccuracy (%p)	-	+0.66	+1.45	+2.01
Precision (%)	94.92	95.48	96.29	96.79
Recall (%)	94.37	95.10	95.74	96.18
F1-score (%)	94.64	95.28	96.01	96.47

**Table 7:** Computational complexity comparison on the Kvasir-v2 Dataset.

Metric	ConvN	ConvV2	EN2	ViT	AMFF-Net (Proposed)
Accuracy (%)	92.95	94.71	92.43	93.61	96.72
Parameters (M)	87.52	87.52	52.46	85.61	88.81
GFLOPs	15.35	15.35	5.22	16.85	16.12
GPU Memory (MB)	1201.86	1355.48	1188.66	1573.80	1472.35
Inference Time (ms/image)	11.98	12.26	9.36	13.87	13.02

## Data Availability

The Kvasir-v2 dataset is available at https://www.kaggle.com/datasets/plhalvorsen/kvasir-v2-a-gastrointestinal-tract-dataset (accessed on 24 March 2026). The HAM10000 dataset is available at https://www.kaggle.com/datasets/kmader/skin-cancer-mnist-ham10000 (accessed on 17 March 2026). The ChestXray14 dataset is available at https://www.kaggle.com/datasets/nih-chest-xrays/data (accessed on 17 March 2026).

## Abbreviations

The following abbreviations are used in this manuscript:

AI	Artificial Intelligence
AMFF	Adaptive Multi-Layer Feature Fusion
AMFF-Net	Adaptive Multi-Layer Feature Fusion Network
AMP	Automatic Mixed Precision
AUC	Area Under the Receiver Operating Characteristic Curve
BCE	Binary Cross-Entropy
BCEWithLogitsLoss	Binary Cross-Entropy with Logits Loss
BiFPN	Bidirectional Feature Pyramid Network
CAD	Computer-Aided Diagnosis
CBAM	Convolutional Block Attention Module
CNN	Convolutional Neural Network
ConvN	ConvNeXt
ConvV2	ConvNeXtV2
CT	Computed Tomography
CUDA	Compute Unified Device Architecture
ECA	Efficient Channel Attention
ECA-Net	Efficient Channel Attention Network
EN2	EfficientNetV2
FA	Feature Alignment
FN	False Negative
FPN	Feature Pyramid Network
FP	False Positive
FPR	False Positive Rate
GAP	Global Average Pooling
GELU	Gaussian Error Linear Unit
GFLOPs	Giga Floating-Point Operations
GRN	Global Response Normalization
HAM10000	Human Against Machine with 10,000 Training Images
MAE	Masked Autoencoder
MRI	Magnetic Resonance Imaging
NIH	National Institutes of Health
PANet	Path Aggregation Network
ROC	Receiver Operating Characteristic
RGB	Red, Green, and Blue
SE-Net	Squeeze-and-Excitation Network
TN	True Negative
TP	True Positive
TPR	True Positive Rate
UNet++	Nested U-Net
ViT	Vision Transformer
XAI	Explainable Artificial Intelligence
